# Supplementary material for: Systemic impacts of disruptions at maritime chokepoints
Source: Nat Commun. 2025 Nov 24;16:10421. doi: 10.1038/s41467-025-65403-w (PMC12644514; doi:10.1038/s41467-025-65403-w)
Supplement: Supplementary file 1 — Supplementary Information [file 41467_2025_65403_MOESM1_ESM.pdf]

Supplementary Figures

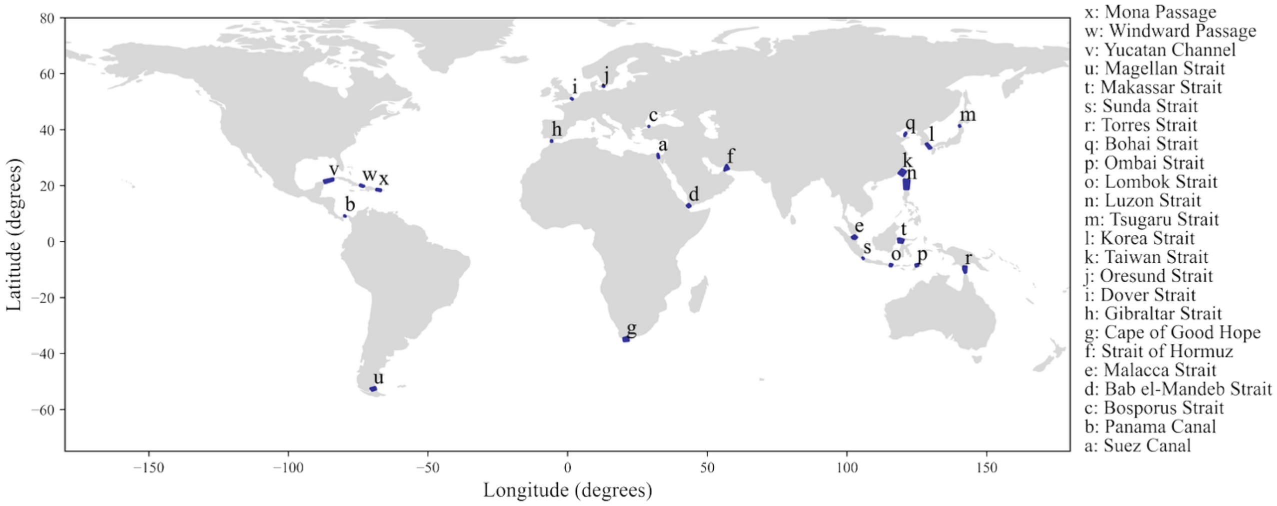

Supplementary Figure 1. Geographical location of the 24 chokepoints considered. The basemap is from GADM (gadm.org).

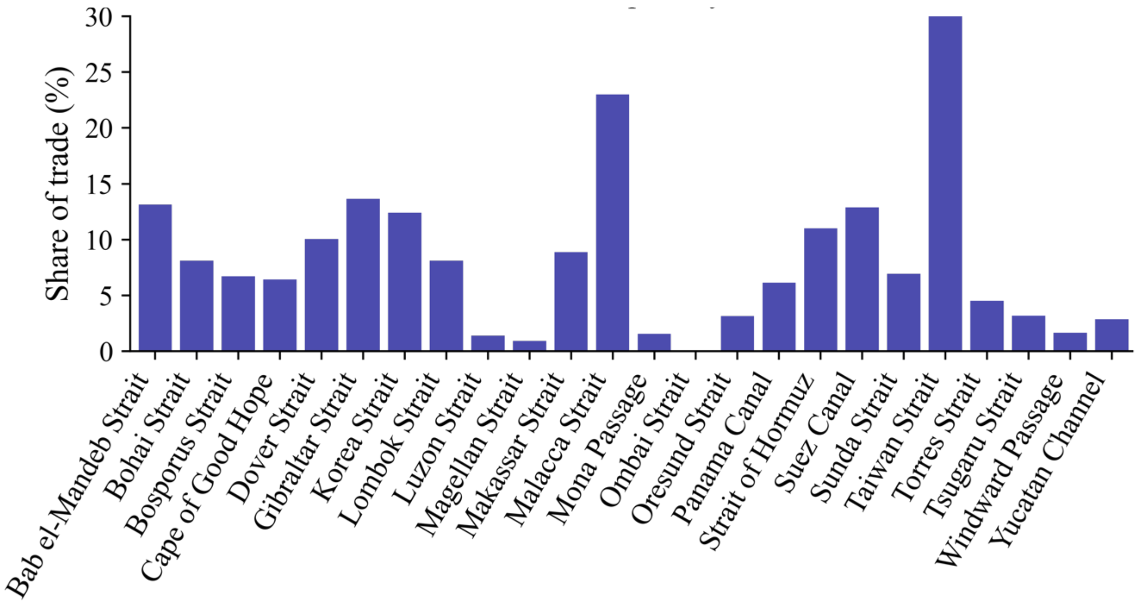

Supplementary Figure 2. Share of global trade volume handled by different chokepoints.

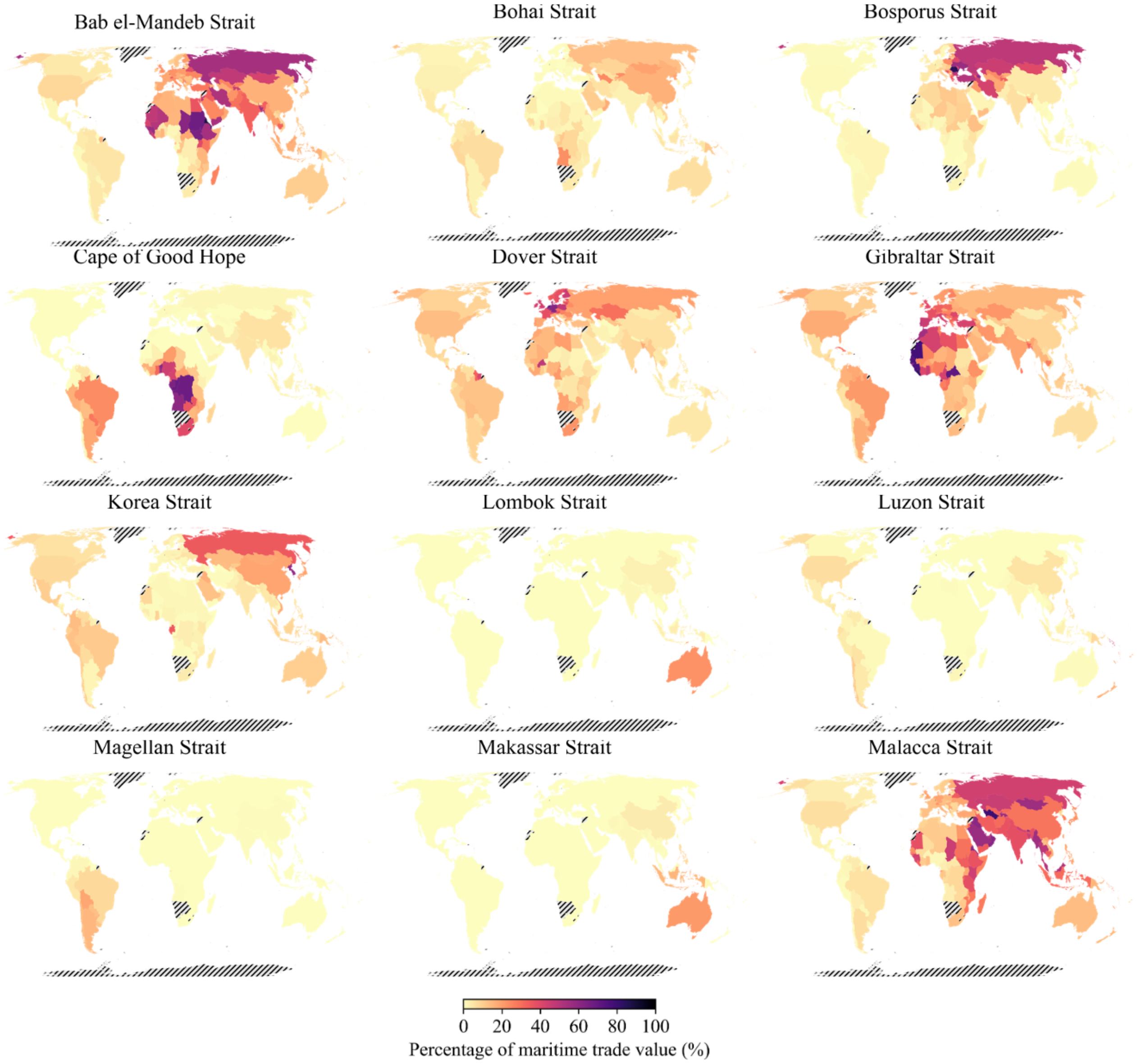
Bab el-Mandeb Strait
Bohai Strait
Bosporus Strait
Cape of Good Hope
Dover Strait
Gibraltar Strait
Korea Strait
Lombok Strait
Luzon Strait
Magellan Strait
Makassar Strait
Malacca Strait
0 20 40 60 80 100
Percentage of maritime trade value (%)

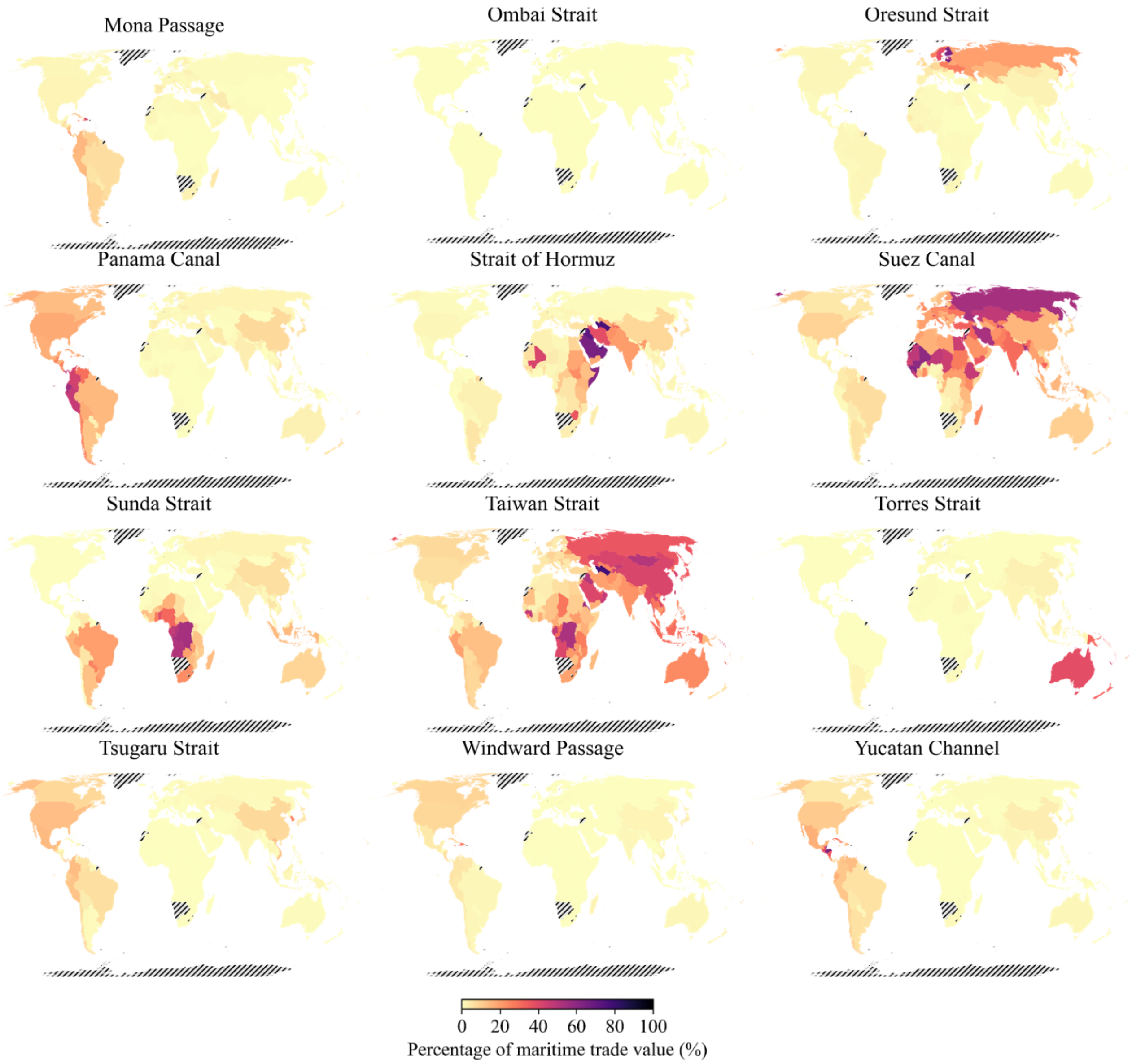

Supplementary Figure 3. Country dependencies on specific chokepoints. The share of maritime trade value flowing through the different chokepoints. The basemap is from GADM (gadm.org).

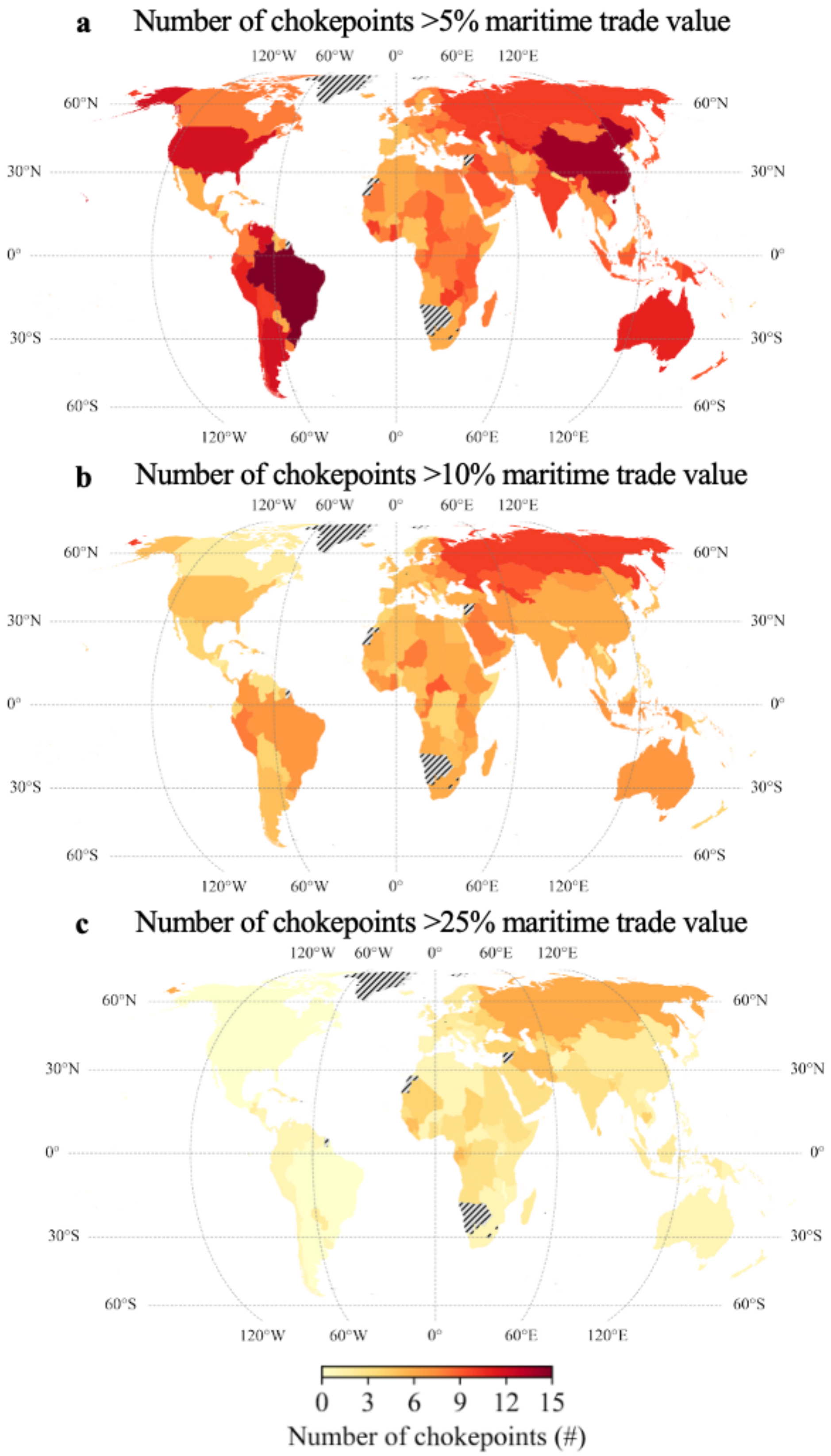

Supplementary Figure 4. Major chokepoint dependencies. Countries' major chokepoint dependencies, defined as facilitating 5% (a), 10% (b) or 25% (c) of a country's maritime trade value. Figures (a), (b) and (c) show the number of chokepoints, out of the 24 considered, that meet this criteria. The basemap is from GADM (gadm.org).

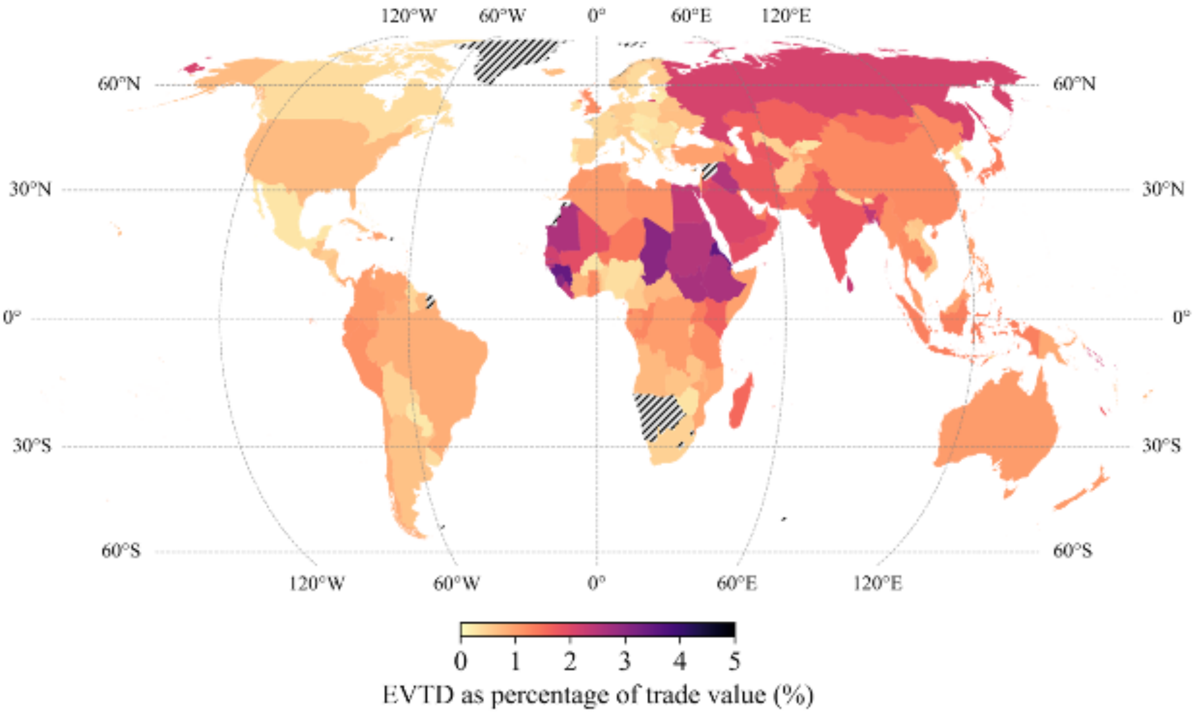

Supplementary Figure 5. EVTD as percentage of total trade value. The basemap is from GADM (gadm.org).

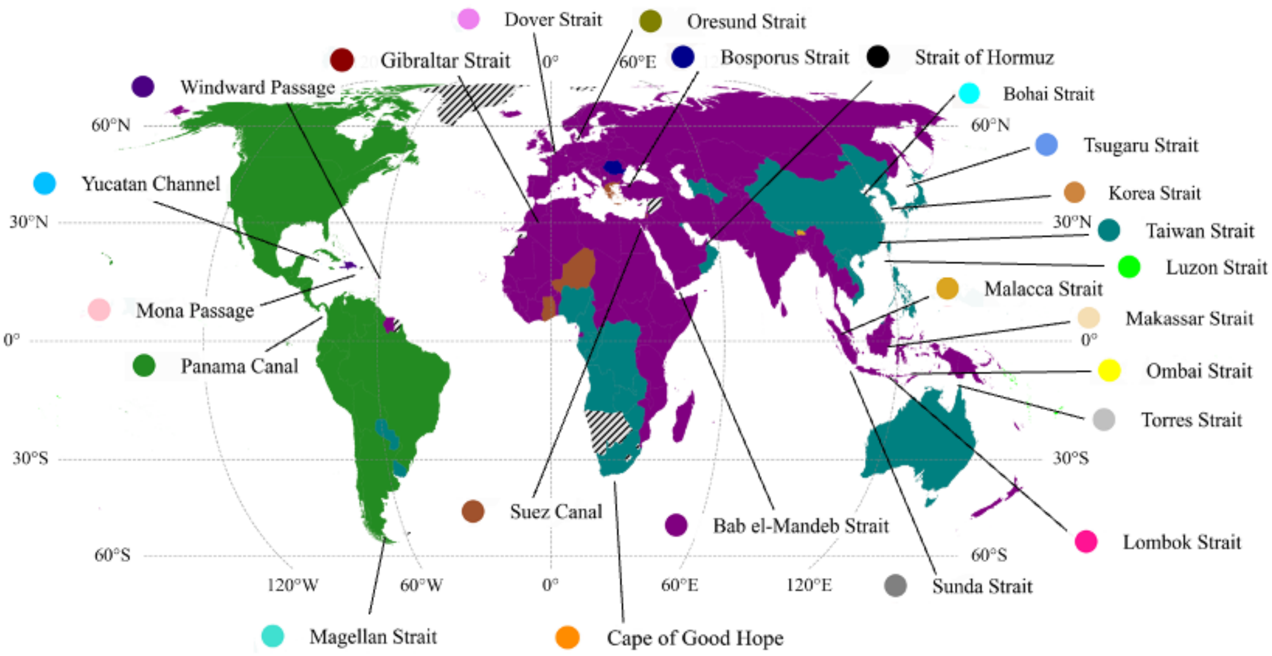

Supplementary Figure 6. Dominant chokepoint of country EVTD. The basemap is from GADM (gadm.org).

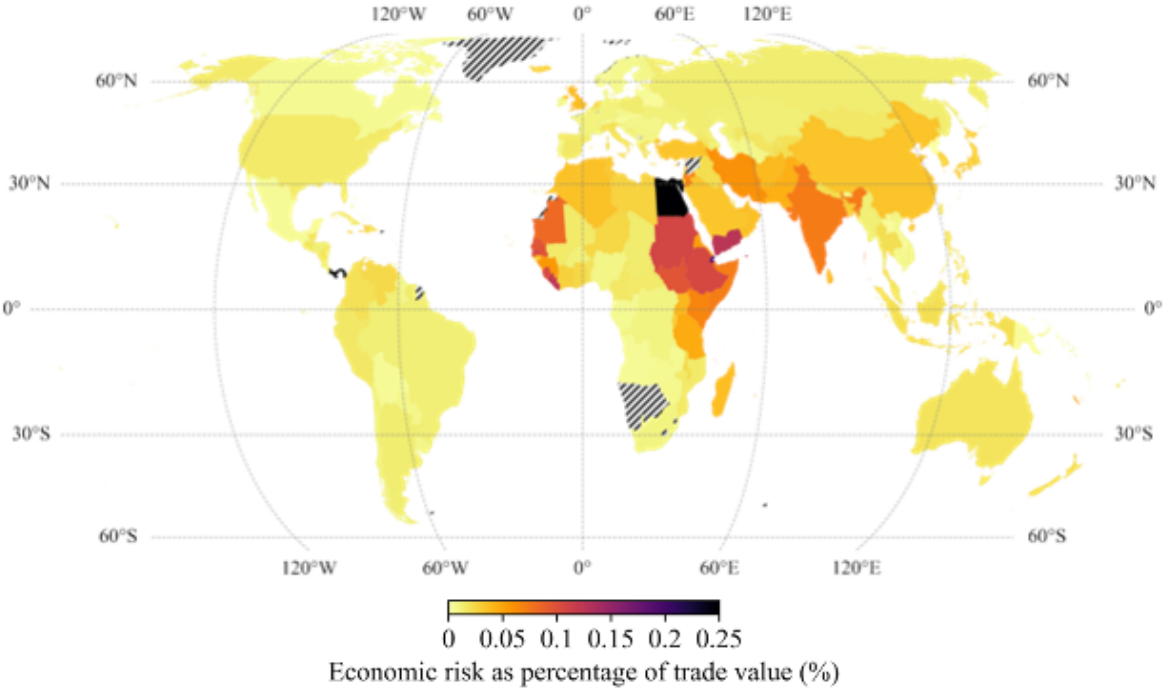

Supplementary Figure 7. Economic risk as percentage of maritime trade value. The basemap is from GADM (gadm.org).

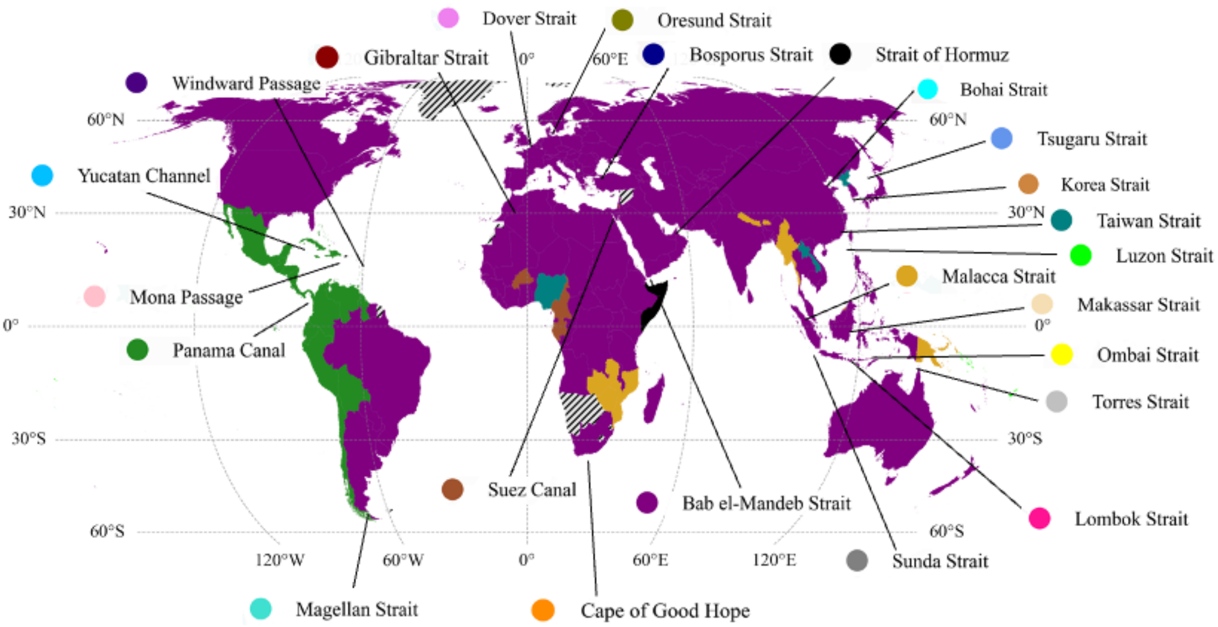

Supplementary Figure 8. Dominant chokepoint of country economic risk. The basemap is from GADM (gadm.org).

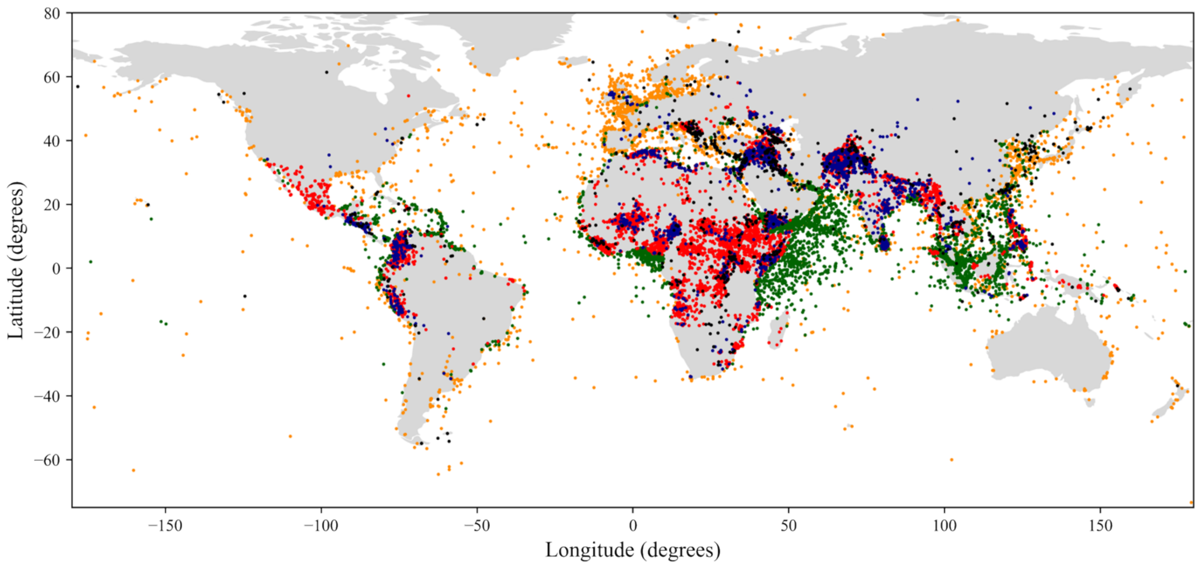

Supplementary Figure 9. The spatial event database of maritime chokepoint hazards. The geographical location of different human-induced hazard events considered, including (i) piracy (green), (ii) maritime incidents (orange), (iii) terrorist attacks (blue), (iv) armed conflict (red), and (v) interstate conflict (black). The basemap is from GADM (gadm.org).

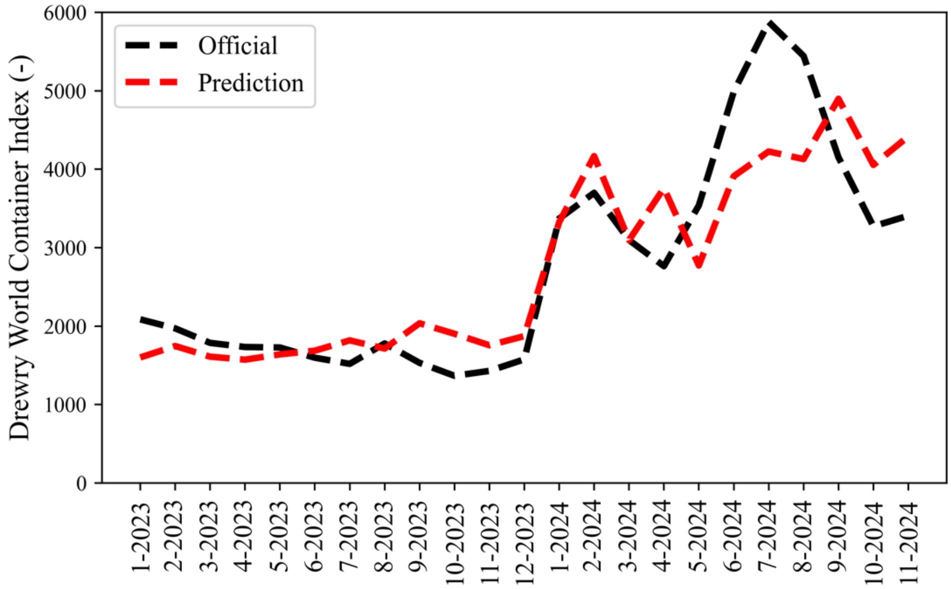

Supplementary Figure 10. Predictive model of container freight rates. Comparison of official and predicted Drewry World Container Index values over the period 2023-2024.

Supplementary Table 1. Global economic risk due to shipping freight spikes causing shipping costs to increase.

**Chokepoint**	**Shipping cost (million USD/yr)**	**Share total (%)**
Bab el-Mandeb Strait	1455.6	42.8%
Bohai Strait	0.0	0.0%
Bosporus Strait	0.0	0.0%
Cape of Good Hope	0.0	0.0%
Dover Strait	3.4	0.1%
Gibraltar Strait	0.6	0.0%
Korea Strait	40.6	1.2%
Lombok Strait	0.8	0.0%
Luzon Strait	27.6	0.8%
Magellan Strait	0.0	0.0%
Makassar Strait	0.0	0.0%
Malacca Strait	158.0	4.6%
Mona Passage	2.9	0.1%
Ombai Strait	8.7	0.3%
Oresund Strait	0.0	0.0%
Panama Canal	279.3	8.2%
Strait of Hormuz	0.0	0.0%
Suez Canal	1293.5	38.0%
Sunda Strait	2.5	0.1%
Taiwan Strait	113.3	3.3%
Torres Strait	0.0	0.0%
Tsugaru Strait	0.9	0.0%
Windward Passage	7.5	0.2%
Yucatan Channel	8.7	0.3%
**Total**	3403.9	100%

Supplementary Table 2. Maritime chokepoints with statistically significantly related hazards leading to compound events.

Maritime chokepoint	Hazard A	Hazard B
Bosporus Strait	Armed conflict	Terrorism
Bab el-Mandeb Strait	Armed conflict	Terrorism
Lombok Strait	Armed conflict	Terrorism

Supplementary Table 3. Maritime chokepoints with statistically significantly related hazards leading to compound events.

Maritime chokepoint A	Maritime chokepoint B	Hazard A	Hazard B
Suez Canal	Strait of Hormuz	Piracy	Piracy
Suez Canal	Strait of Hormuz	Armed conflict	Piracy
Panama Canal	Strait of Hormuz	Piracy	Piracy
Panama Canal	Bab el-Mandeb	Piracy	Piracy
Malacca Strait	Bab el-Mandeb	Piracy	Piracy
Strait of Hormuz	Bab el-Mandeb	Piracy	Piracy
Sunda Strait	Bab el-Mandeb	Piracy	Piracy

Supplementary Table 4. List of combinations of maritime chokepoints affected by the same tropical cyclone.

Chokepoint combination	Number of occurrences	Size	Likelihood of return (in yrs)
(Luzon Strait, Taiwan Strait)	14083	2	0.71
(Mona Passage, Windward Passage)	2851	2	3.51
(Korea Strait, Luzon Strait)	1026	2	9.75
(Korea Strait, Tsugaru Strait)	928	2	10.78
(Windward Passage, Yucatan Channel)	779	2	12.84
(Korea Strait, Taiwan Strait)	686	2	14.58
(Mona Passage, Yucatan Channel)	635	2	15.75
(Bohai Strait, Korea Strait)	571	2	17.51
(Luzon Strait, Tsugaru Strait)	218	2	45.87
(Bohai Strait, Taiwan Strait)	152	2	65.79
(Bohai Strait, Luzon Strait)	140	2	71.43
(Taiwan Strait, Tsugaru Strait)	130	2	76.92
(Gibraltar Strait, Mona Passage)	9	2	1 111.11
(Lombok Strait, Ombai Strait)	8	2	1 250.00
(Gibraltar Strait, Windward Passage)	5	2	2 000.00
(Mona Passage, Panama Canal)	4	2	2 500.00
(Gibraltar Strait, Yucatan Channel)	2	2	5 000.00
(Panama Canal, Windward Passage)	2	2	5 000.00
(Korea Strait, Luzon Strait, Taiwan Strait)	568	3	17.61
(Mona Passage, Windward Passage, Yucatan Channel)	510	3	19.61
(Korea Strait, Luzon Strait, Tsugaru Strait)	192	3	52.08
(Korea Strait, Taiwan Strait, Tsugaru Strait)	122	3	81.97
(Bohai Strait, Luzon Strait, Taiwan Strait)	120	3	83.33
(Luzon Strait, Taiwan Strait, Tsugaru Strait)	106	3	94.34
(Gibraltar Strait, Mona Passage, Windward Passage)	3	3	3 333.33
(Mona Passage, Panama Canal, Windward Passage)	1	3	10 000.00
(Korea Strait, Luzon Strait, Taiwan Strait, Tsugaru Strait)	100	4	100.00

Supplementary Table 5. Number of cyclones hitting a chokepoint across the 10,000 years of synthetic cyclone events.

Chokepoint	Number of events	Annual hit rate
Bab el-Mandeb Strait	35	0.004
Bohai Strait	2453	0.245
Bosporus Strait	0	0.000
Cape of Good Hope	4	0.000
Dover Strait	10	0.001
Gibraltar Strait	288	0.029
Korea Strait	10429	1.043
Lombok Strait	111	0.011
Luzon Strait	30101	3.010
Magellan Strait	0	0.000
Makassar Strait	0	0.000
Malacca Strait	0	0.000
Mona Passage	6496	0.650
Ombai Strait	135	0.014
Oresund Strait	0	0.000
Panama Canal	102	0.010
Strait of Hormuz	96	0.010
Suez Canal	0	0.000
Sunda Strait	5	0.001
Taiwan Strait	17967	1.797
Torres Strait	371	0.037
Tsugaru Strait	3598	0.360
Windward Passage	4492	0.449
Yucatan Channel	3737	0.374

Supplementary Table 6. Three different trade groups and corresponding inventory levels.

Group	Fraction	Inventory days
1	0.15	7
2	0.50	25
3	0.35	60
